# Basin-Scale Control on the Phytoplankton Biomass in Lake Victoria, Africa

**DOI:** 10.1371/journal.pone.0029962

**Published:** 2012-01-09

**Authors:** Andrés Cózar, Miguel Bruno, Nadia Bergamino, Bárbara Úbeda, Luca Bracchini, Arduino M. Dattilo, Steven A. Loiselle

**Affiliations:** 1 Departamento de Biología, Universidad de Cádiz, Puerto Real, Cádiz, Spain; 2 Centro Andaluz de Ciencia y Tecnología Marinas (CACYTMAR), Universidad de Cádiz, Puerto Real, Cádiz, Spain; 3 Environmental Spectroscopy Group, Dipartimento Farmaco Chimico Tecnologico, Università di Siena, Siena, Italy; 4 Consorzio Interuniversitario per lo Sviluppo dei Sistemi a Grande Interfase (CSGI), Firenze, Italy; Institute of Marine Research, Norway

## Abstract

The relative bio-optical variability within Lake Victoria was analyzed through the spatio-temporal decomposition of a 1997–2004 dataset of remotely-sensed reflectance ratios in the visible spectral range. Results show a regular seasonal pattern with a phase shift (around 2 months) between the south and north parts of the lake. Interannual trends suggested a teleconnection between the lake dynamics and El-Niño phenomena. Both seasonal and interannual patterns were associated to conditions of light limitation for phytoplankton growth and basin-scale hydrodynamics on phytoplankton access to light. Phytoplankton blooms developed during the periods of lake surface warming and water column stability. The temporal shift apparent in the bio-optical seasonal cycles was related to the differential cooling of the lake surface by southeastern monsoon winds. North-south differences in the exposure to trade winds are supported by the orography of the Eastern Great Rift Valley. The result is that surface layer warming begins in the northern part of the lake while the formation of cool and dense water continues in the southern part. The resulting buoyancy field is sufficient to induce a lake-wide convective circulation and the tilting of the isotherms along the north-south axis. Once surface warming spreads over the whole lake, the phytoplankton bloom dynamics are subjected to the internal seiche derived from the relaxation of thermocline tilting. In 1997–98, El-Niño phenomenon weakened the monsoon wind flow which led to an increase in water column stability and a higher phytoplankton optical signal throughout the lake. This suggests that phytoplankton response to expected climate scenarios will be opposite to that proposed for nutrient-limited great lakes. The present analysis of remotely-sensed bio-optical properties in combination with environmental data provides a novel basin-scale framework for research and management strategies in Lake Victoria.

## Introduction

The world's great lakes constitute vital ecosystems with complex conservation challenges.While high spatio-temporal variability is often shown in these massive ecosystems [1]–[3], specific basin-scale mechanisms controlling biological and biogeochemical variability have yet to be thoroughly explored [4]. Understanding the spatio-temporal dynamics and their relationship with the environmental drivers is a basic step in establishing effective strategies for the management of the great lakes. However, this task often lacks suitable data at basin and long-term scales. This shortcoming is rooted in the mismatch between the scales of ship-borne research and planktonic variability [4]. Financial and logistic constraints hamper surveys which combine both high sampling frequency and extensive spatial measurements.

Satellite-based sensors allow for the observation of extensive areas with a high frequency, representing the only realistic way to study many processes at basin-wide scale. Remote-sensed estimates of phytoplankton biomass have led to fundamental insights in marine ecosystems [5]–[6]. However, general calibration algorithms have yet to be developed for inland waters, where non-algal water components often play an important role in the optical properties [7]. Nevertheless, existing long time series of remote-sensed reflectance can provide a valuable opportunity to advance the basin-scale knowledge of the most productive great lakes. Phytoplankton dynamics play a major role in the optical properties of these extensive lakes because of the relatively limited influence of water components of terrestrial origin over much of the lake surface.

Lake Victoria is the largest tropical lake and the world's second largest lake. It constitutes a massive conduit of carbon between land and atmosphere [8], a reservoir of global biodiversity [9] as well as a fundamental source of revenue and nutrition for the three riparian countries [10]. Likewise, Lake Victoria represents a remarkable example of the possible human impacts on the biological and biogeochemical functioning of the large lakes [11]. During the last decades, a progressive eutrophication has resulted from increased phosphorous loads, mainly due changing demographics and resource use patterns in the drainage basin [11]–[12]. This has been accompanied by a profound shift in the phytoplankton composition. Diazotrophic cyanobacteria, which have the ability to fix atmospheric nitrogen, have become dominant [13]. As a result, phytoplankton biomass in Lake Victoria has reached concentrations that are unlikely to be found among world's large lakes [8], [14]. Light availability for phytoplankton production has become greatly reduced because of self-shading [15]. Light is now limiting nitrogen fixation and constrains phytoplankton production and biomass over much of lake and throughout much of the year [16], [17].

Limnological research in Lake Victoria, some dating from the beginning of the last century, has been highly fruitful. However, intensive monitoring has been generally focused on a single year and often limited to the northern part of the lake [15], [16], [18]. Field studies reveal that the complete mixing of the water column occurs at least once a year [18]. As vertical mixing depth reduces light availability for phytoplankton, a seasonal peak of phytoplankton biomass occurs during the stratification period [13], [15]. However, the lack of appropriate datasets makes it difficult to assess if this seasonal cycle shows similar periodicity across the lake or how the phytoplankton dynamics is affected by the year-to-year environmental variability.

Among the world's largest lakes, Lake Victoria is remarkably suited for tracking phytoplankton dynamics by the use of remote sensors. It shows the highest chlorophyll concentrations of any great lake [14]. Additionally, most of the water input to the lake (around 80%) is from direct rainwater [19], which limits the impact of optically active water components of terrestrial origin. Therefore, light attenuation in most of the lake is strongly linked to phytoplankton [17]. In the present study, we analyze the seasonal and interannual patterns of remotely-sensed optical variability from 1997 to 2004 with the aim of describing the basin-scale phytoplankton dynamics in Lake Victoria. These temporal patterns are then analyzed in relation to regional and global environmental drivers in order to identify specific large-scale regulatory mechanisms for the phytoplankton dynamics.

## Methods

Maximum band ratios (*MBR*) are commonly used to estimate phytoplankton biomass (measured as chlorophyll-a concentration, *chl*) with satellite color sensors [20]. *MBR* are determined from the maximal ratio between a reference reflectance (*R_rs_^λi^*) measured at a wavelength in the green domain of the spectrum (*λ_i_*∼550–560 nm), and one of three available wavelengths (*R_rs_* ^λ*j*^) in the blue and blue-green domain (*λ_j_*∼443, ∼490 or ∼510 nm). The maximal value of the three possible blue-to-green ratios decreases with increasing *chl* while maintaining a high sensor signal-to-noise ratio [21]. Calibration algorithms between *MBR* and *chl* have been developed for extensive ocean datasets. In inland waters, the concentrations of chromophoric dissolved organic matter (CDOM) and non-algal particles are often considerably higher than in ocean ecosystems, hindering the application of the ocean-based calibration algorithms. A relevant presence of these components leads to reduction of the *MBR* measurements and an overestimate of *chl* [7]. The occurrence of mineral sediments or terrestrial CDOM which do not co-vary with *chl* is especially worrying for calibration purposes, but direct correlation between *MBR* and *chl* is feasible in inland waters where optical properties are dominated by phytoplankton and associated components such as algal derived CDOM or detrital particles. In the extensive offshore waters of large lakes, resuspended sediment, river discharges or wetland released CDOM release play a limited role on the water optical properties in relation to phytoplankton biomass [22]. Therefore, while specific *MBR*-*chl* calibrations generally remain unresolved for great lakes, *MBR* can provide information on the spatio-temporal dynamics in waters where the phytoplankton controls the variability of the optical properties. In this regard, eutrophic Lake Victoria is particularly suitable due to the influence of *chl* on the underwater light extinction [17].

In the present study, we analyze the relative variability of *MBR* in offshore areas of Lake Victoria. The primary data were normalized water-leaving radiances [23] retrieved by the SeaWiFS platform (http://oceancolor.gsfc.nasa.gov). In turbid waters, *R_rs_* ^510^/*R_rs_* ^555^ is the most probable maximum band ratio for the SeaWiFS sensor [21]. In this study, *MBR* was based on *R_rs_* ^510^/*R_rs_* ^555^ for the 95% of the data examined. It should be noted that other ocean color sensors such as MODIS do not cover this blue-green domain (510 nm) of the spectrum.

SeaWiFS-derived *MBR* decrease exponentially with increasing *chl* [21]. Therefore, variability at low phytoplankton concentrations is magnified while variability is reduced for high concentrations. To overcome this uneven treatment of optical variability, we transformed *MBR* by using the OC4v4 algorithm for *chl* [21]. This calibration algorithm is based on a polynomial of log-transformed *MBR* which linearly correlates with the log-transformed *chl*. In this document, we will refer to the OC4v4-transformed *MBR* as chlorophyll concentration (OC4v4-*chl*) although this bio-optical variable also depends on other optically-active components.

The present study covers the period from September 1997 to March 2004. Data corresponding to a 10-km buffer between the open lake and the coast were removed from the dataset to avoid local sources of variability related to terrestrial or sedimentary origin [22]. The masked areas have the highest *chl* [17] as phytoplankton concentrations increase with shoaling bottom depths in light limited ecosystems [24]. Nevertheless, they are less sensitive to basin-scale changes in mixing depth because, being shallow areas (e.g. <20 m), the mixing depth usually reaches the lake bottom [11].

Temporal and spatial patterns in the offshore dataset were determined through Single Value Decomposition (SVD; [25]). As SVD must be applied to gap-free time series (without missing data), data smoothing in time and space was required to eliminate gaps in the dataset related to cloud cover. Thus, we used bi-weekly 9×9 km averages of the original daily 1×1 km SeaWiFS imagery. This smoothing level permits the analysis of basin-scale variability over seasonal or longer time scales. Remaining gaps (<10%) were filled through spatial interpolation following a kriging approach. The complete OC4v4-*chl* matrix consisted of 150 time-scenes, each containing 594 lake-sections.

SVD provides a series of eigenfunctions associated with dominant temporal modes of the OC4v4-*chl* variability in the entire dataset. For each eigenfunction, the related eigenvalue indicates the fraction of the total variability explained. Significant temporal modes determined by SVD were then compared to the local OC4v4-*chl* time series of each lake-section. In this manner, it was possible to associate each lake section to a specific temporal mode, based on the highest Pearson correlation coefficient [5]. Lake-sections sharing the same dominant temporal mode were then grouped to delimit co-varying lake regions [26]. Seasonal and interannual components were then extracted from the average OC4v4-*chl* time series of each region by applying an Auto-Regressive Integrated Moving Average model (ARIMA; [27]).

Spatial-averaged estimations of lake surface temperature (*LST*) in the co-varying regions were obtained using AVHRR data (http://podaac-www.jpl.nasa.gov/sst). Monthly temperatures were calculated from the average of the monthly day and night temperatures. Given that the temperature of the deep water in Lake Victoria is fairly constant [18], surface temperature was used to examine temporal patterns of stratification.

**Figure 1:**
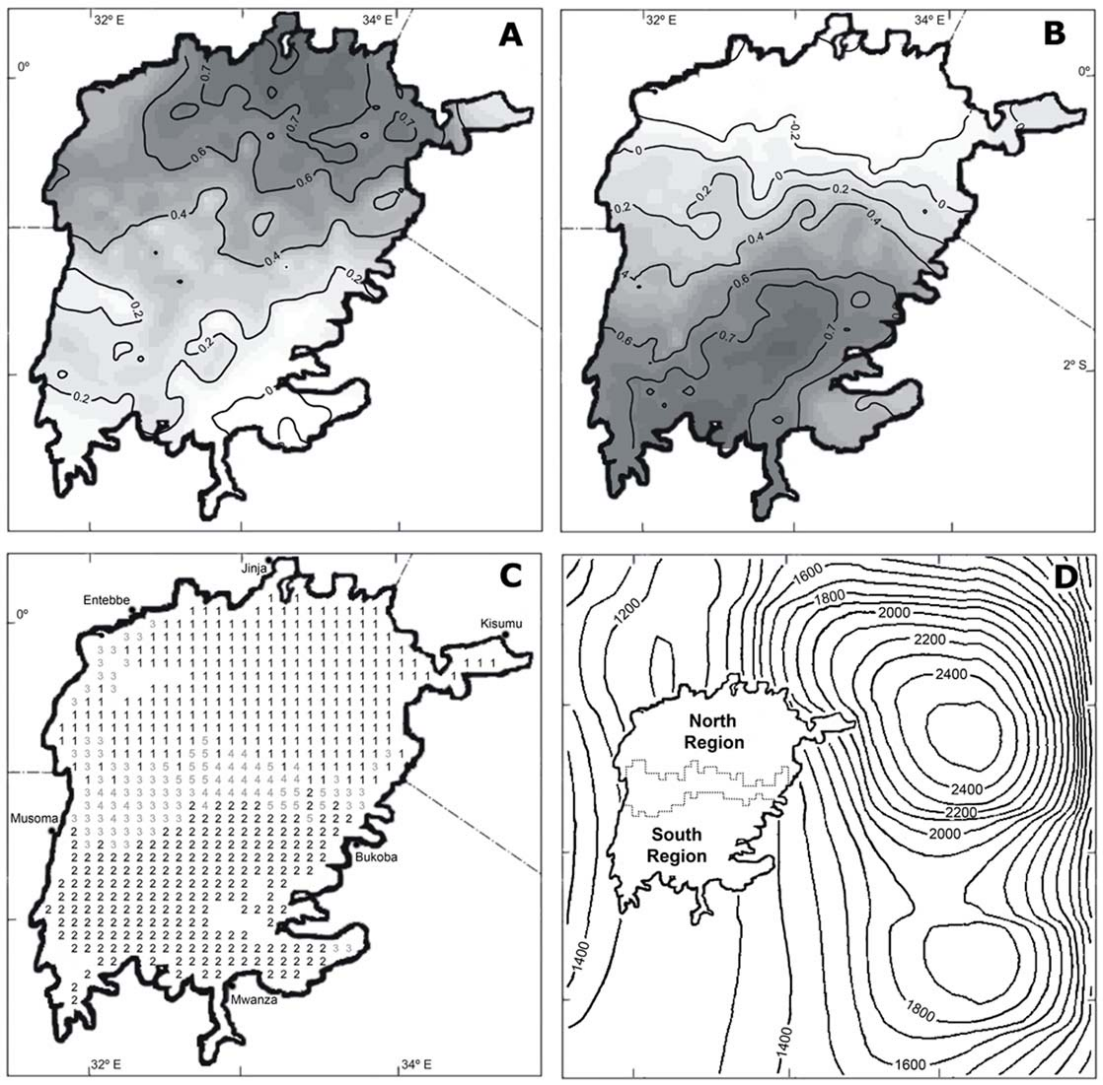
Bio-optically co-varying regions in Lake Victoria. (A) Spatial pattern of the Pearson correlation coefficients between the mode 1 and the OC4v4-*chl* time series of the lake-sections. (B) Spatial pattern of Pearson correlation coefficients between the mode 2 and the OC4v4-*chl* time series. (C) Contour lines were obtained by kriging interpolation. Spatial distribution of the most representative mode (highest R) for each lake-section. (D) Classification of Lake Victoria in regions sharing similar bio-optical co-variation. The Transitional Region between North and South Regions is delimited by dotted lines. The topography of the study area is also shown through the contour heights (in meters) interpolated from a 10' dataset.

The seasonal changes in water column stability were also explored by determining the net heat flux (*NHF*) into the lake surface layer. *NHF* was estimated by the following energy balance:
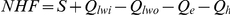
where *S*, *Q_lwi_*, *Q_lwo_*, *Q_e_* y *Q_h_* are respectively the monthly heat fluxes due to incident solar radiation, incoming long wave radiation from the atmosphere, outcoming long wave radiation to the atmosphere, latent heat of vaporization and sensible heat. Data of cloud cover, vapor pressure and air temperature were compiled from the Department of Water Resources Management of Uganda, the Kenyan Marine Fisheries Research Institute and the Tanzania Meteorological Agency for six shoreline stations around the lake (Entebbe, Jinja, Kisumu, Musoma, Mwanza and Bukoba; Fig. 1C). Proceeding from the assumption that lake regions with similar bio-optical variability also have similar driving forces, meteorological data of the stations bordering the same region were averaged to determine mean time series for each lake region. It should be noted that shore-based wind data may be subject to local land-lake effects [28]. Unfortunately, wind data collected over Lake Victoria are still very limited [29]. Regional wind data from National Center for Environmental Prediction (NCEP) reanalysis [30] was used to model the basin-scale wind field. Annual mean wind speed (1.8 m s^−1^) over Lake Victoria derived from NCEP reanalysis agrees with the mean of approximately 2 m s^−1^ derived from ten stations over the open lake and bays [29]. Before estimating heat fluxes, a Fast Fourier transform filter was applied to these meteorological time series in order to remove fluctuations with periods shorter than the seasonal cycle. For *S*, a mean historical seasonal cycle [31] was used. The remaining surface energy fluxes were estimated using the following algorithms [32]:
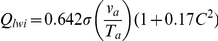


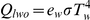


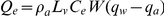


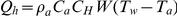
where *σ* = 5.67·10^−8^ W K^−4^ m^−2^ is the Stefan-Boltzmann constant; *v_a_* is the vapour pressure; *T_a_* is the air temperature; *C* is the cloud cover fraction; *e_w_* = 0.97 is the emissivity of the water surface; *T_w_* is the temperature at the water surface estimated from AVHRR data (*LST*); *ρ_a_* = 1.3 Kg m^−3^ is the air density; *L_v_* = 2256 KJ Kg^−1^ is the latent heat of vaporization; *C_e_* and *C_H_* are respectively the latent heat and sensible heat transfer coefficients at 10 m (*C_e_* = *C_H_* = 2.5 10^−3^ [32]); *W* is the wind speed at 10 m height; *q_w_* is the specific humidity at saturation pressure at *T_w_*; *q_a_* is the specific humidity of the air; *C_a_* is the specific heat of air.

## Results

The decomposition of the OC4v4-*chl* variability from 1997 to 2004 in Lake Victoria provided five significant temporal modes (of a total of 149) which account for 63.5% of the total variability. The first two modes accounted for most of the variability (48.6%). Specifically, the variance explained by the modes 1 and 2 were 31.1% and 17.5% respectively. These two modes were found to correlate with the temporal variability of OC4v4-*chl* over extensive areas of the lake (Fig. 1A and 1B). OC4v4-*chl* variability of the northern part of the lake was found to correlate best with mode 1, while mode 2 was found to characterize the southern part. Modes 3, 4 and 5 were found to characterize small coastal areas (Inner Speke Gulf in the southeast and the area protected by Sese Islands in the northwest) and the central band separating the northern and southern half of the lake (Fig. 1C). This temporal variability allowed us to divide Lake Victoria into three large regions; North, South and Transitional Regions (Fig. 1D). While near coast areas in the North and South Regions were associated with modes 3, 4 and 5, significant correlations with modes 1 and 2 also occurred. The Transitional Region did not have a significant correlation with modes 1 and 2, and was best correlated to the modes 3, 4 and 5.

**Figure 2:**
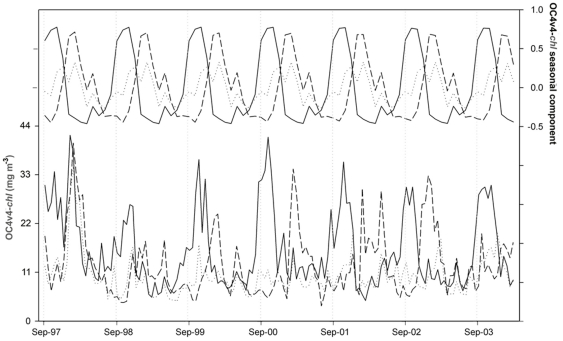
Bio-optical variability in Lake Victoria for the 1997–2004 period. OC4v4-*chl* time series for different lake Regions (bottom) and their corresponding seasonal components (top). North Region, solid line; Transitional Region, dotted line; South Region, dashed line.

The regional OC4v4-*chl* time series generally reached higher peaks in the North Region than in the South Region (Fig. 2). Time-averaged OC4v4-*chl* was a 25% higher in the North Region. We also found a persistent temporal shift between the annual seasonal maxima of the two major regions in all years except in the 1997–98 cycle. The seasonal component of the North Region shows a maximum in October–November, while the seasonal maximum in the South Region occurs in January–February. A second smaller peak also occurs around April in both regions. This second seasonal peak is considerably higher in the South Region. The Transitional Region showed an intermediate pattern between North and South. This may be related to mobility in the regional limits of North and South Regions. It is important to note that the spatial limits of the regionalization are a simplification of the basin-scale heterogeneity.

**Figure 3:**
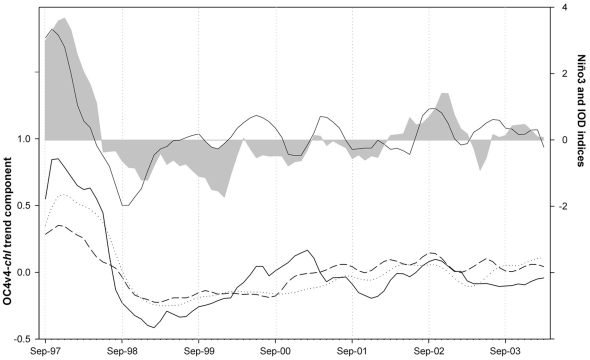
Interannual bio-optical variability in Lake Victoria for 1997–2004 and global indices of interannual climate fluctuations. Trend components extracted from the OC4v4-*chl* series (bottom). North Region, solid line; Transitional Region, dotted line; South Region, dashed line. Indices of interannual climate variability linked to ENSO (shaded area) and IOD (solid line) phenomenon (top). Linear correlations between trend components and climate indices were statistically significant at the 99% confidence level for all the combinations (R>0.840, p<0.001, n = 79).

The regional time series showed a high interannual variability in OC4v4-*chl*, with particularly high values found in the 1997–98 cycle. In this year, the annual mean was 39% higher in the South Region and 45% higher in the North Region with respect to the average of the other years. OC4v4-*chl* also increased faster in North Region than in South Region in this cycle, but the annual maximum was simultaneously reached in January in both regions (Fig. 2). By extracting the interannual trend components from the regional series, it was possible to make a direct comparison with the variability of interannual climate global indices (Fig. 3). A clearly synchronous behavior was found between ENSO (El Niño Southern Oscillation) index, IOD index (Indian Ocean Dipole) and the bio-optical trends. This relationship suggests an important role of climate forcing on the dynamics of Lake Victoria.

**Figure 4:**
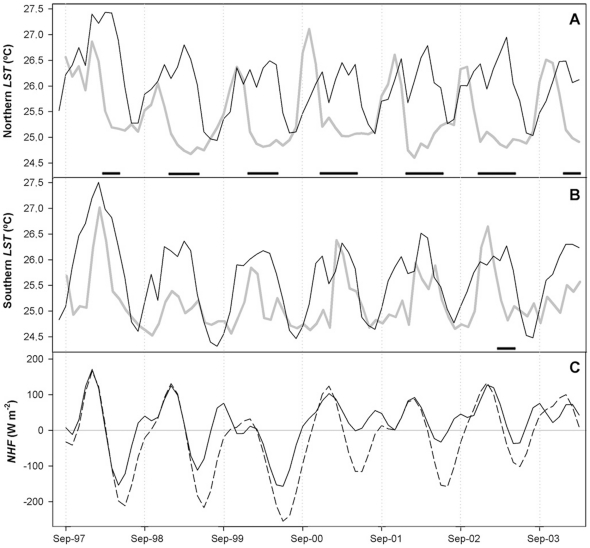
Temporal variability of water temperature (*LST*) and net heat flux (*NHF*) in the surface layer of Lake Victoria. *LST* time series for North Region (A) and South Region (B) indicated by a solid line. The grey line represents the corresponding monthly variability of OC4v4-*chl* for each Region. Scaling is the same for both graphs (0–44 mg m^−3^). (C) *NHF* into the surface layer in the North (solid line) and South (dashed line) Regions. NHF was significantly related with the monthly *LST* variation (*LST_t_ – LST_t−1_*) (R = 0.651, p<0.001, n = 158). Black bars in the lower axes of figures A and B indicate periods of bloom collapse during the seasonal warm phase.

Lake surface temperature (*LST*) showed a regular seasonal pattern (Fig. 4). The seasonal timing of *LST* was similar for the South and North Regions and consistent with the cycles of vertical thermal stratification observed in past studies for the northern part of the lake [18], [33]. A warming period, from September to December, was followed by a period of high surface temperature corresponding to the stratification phase, from January to May. In the mixing phase, between June and August, surface temperatures fell to their annual minimum values.

Net heat fluxes (*NHF*) across the lake surface were in agreement with *LST* dynamics except during the onset of the heat gain period in the South Region, which had a two months delay with respect to the *LST* rise. Relative differences also emerge from the comparison of the seasonal amplitudes of *LST* and *NHF* in the two regions. *LST* remains lower in the south with respect to the north, especially in the mixing and warming phase (Fig. S1). Likewise, *NHF* shows wider seasonal amplitude in the South Region as result of the more intense surface cooling during the mixing period. This confirms reports of a stronger mixing in the southern part of the lake [18]. The interannual analysis showed particularly high *LST* in 1997–98. This increase was also observed in the determination of surface heat gain, especially between December and February, in correspondence with the OC4v4-*chl* maxima (Fig. 4).

*NHF* was dominated by the evaporative heat flux (*Q_e_*), which is governed by the wind flow over the lake and related variables such as the vapor pressure. Annual mean *Q_e_* was 104 W m^−2^ for the North Region and 141 W m^−2^ for the South Region. These spatial differences agree with the comparison of the evaporation rates measured in the northern coast (lower) with respect to the southern coast [31].

## Discussion

A stable seasonal pattern of OC4v4-*chl* was found in Lake Victoria for the 1997–2004 period (Fig. 2). The seasonal pattern of the phytoplankton biomass is well known for the northern part of the lake [15]. A comparison of the 1997–2004 averaged seasonal cycle of OC4v4-*chl* in North Region and the 1990–91 seasonal cycle of *chl* measurements performed in this part of the lake reveals a strong correspondence, coinciding maxima and minima of both series (R = 0.790, p = 0.002, n = 12). OC4v4-*chl* depends on the phytoplankton concentration as well as other water components, however, these data support the hypothesis that phytoplankton constitutes the main driver of OC4v4-*chl* variability in the offshore waters (see also Figure S2).

Field studies have shown the linkage between the seasonal cycle of phytoplankton biomass and water column mixing, related to the availability of light during the stratification phase [13]. In light-limited lakes, the development of the phytoplankton blooms often occurs immediately after the seasonal increase in surface water temperature, following the end of the seasonal mixing by convection [34]. In fact, seasonal increases in surface temperature (*LST*) coincide with rises of OC4v4-*chl* in the North Region (Fig. 4A). In the South Region, however, the increase of OC4v4-*chl* is delayed around two months after the onset of the lake surface warming (Fig. 4B). *LST* maintained a similar seasonal timing throughout the lake regions, without displaying the north-south shift apparent in the bio-optical seasonal cycles. On the other hand, *NHF* and OC4v4-*chl* dynamics were clearly coupled in both regions. Additionally, it should be noted that, on average, the southern region acted as heat source to the atmosphere (mean *NHF* = −20.5 W m^−2^) while northern region as heat sink (mean *NHF* = 19.6 W m^−2^). The southern region was also cooler than the northern region (Fig. S1). This spatially distinct heat distribution shows strong similarities with that recently described for Lake Tanganyika, where the differential cooling in the southern and northern basin drives a large-scale convective circulation [35].

Seasonal surface cooling in Lake Victoria is primarily driven by the dominant southeastern monsoon winds, which are, in turn, influenced by the latitudinal migration of the Inter-tropical Convergence Zone (ITCZ) across the region [36]. Between April and September, the ITCZ moves northward as relatively strong southeasterly winds enter the lake from the Indian Ocean [37]. The latitudinal differences of the wind field in Lake Victoria (Fig. S3) can be partly attributed to the passage of the ITCZ across the lake. Nevertheless, several studies emphasize the relevance of orography on the meso-scale wind system of the region [38]–[39]. The topography around the lake shows that the north half of the lake is sheltered from the easterly wind flow by the mountain chains bordering the Eastern Great Rift Valley (Fig. 1D). This would further enhance the north-south difference in cooling by the southeast trade wind flow. Winds are strengthened as they enter the southern passage in the mountain chains, and become weaker and moister as they move northward across the lake.

A large-scale convective circulation in Lake Victoria may be established when, contemporarily, surface water is being warmed in the north while the formation of cool and dense water continues in the south (Fig. 4). This period corresponds to the seasonal weakening of the southeast trade winds around September. In this month, the north-south differences in *LST* were also maximal (Fig. S1). *LST* in the south was lower by 0.7°C compared to the north. A convective circulation implies a downwelling of cooled surface water in the southern region and a deep advection of this cool and dense water towards the north. In the north, the warming of surface water would be linked to the return flow of warmed surface water going southward. This circulation cell implies a southward tilting of the isotherms in the lake. An alternative large-scale circulation pattern could be driven by the wind-induced surface currents. Southeastern surface currents would lead to a circulation which is opposite to that of the convective cell. However, the features of the lake basin and wind flow over the region indicate that the effects of convection dominate over wind-induced currents between August and November (see Figure S4).

The occurrence of a lake-wide convection cell around September offers an explanation for the observed energy and bio-optical patterns. The southward surface warm limb of the circulation explains the timing of the seasonal rises in *LST* across the lake. The resulting heat transfer from the northern to the southern region also explains why the annual net heat exchange with the atmosphere was relatively large and had opposite signs in the two regions, while there was no significant change in the annual *LST* of either region during the study period. Likewise, the period of convective circulation overlaps with the phase shift in the OC4v4-*chl* cycles of the lake regions (Fig. 4). The subduction of the cool limb of the circulation below the surface warm water in the north must increase the vertical density gradients and favor the accumulation of phytoplankton cells in the well-lit surface layer of North Region. At the same time, the surface cooling prevents phytoplankton bloom development in the South Region. The mid-lake boundary found between North and South Regions could mark where cooler water transported northward is driven under warmer water moving southward.

The seasonal phases of high OC4v4-*chl* occur during the warm (stratified) period and do not extend beyond the beginning of the surface cooling (and the deep-water mixing period) in any of the years analyzed. Nevertheless, it should also be noted that OC4v4-*chl* often (especially in North Region) decreases before the end of the warm period, when the conditions are apparently still favorable for the permanence of the phytoplankton in the top of the water column (periods with black bars in Fig. 4). This decline in OC4v4-*chl* may have several causes. However, experiments on the effect of the grazing or iron limitation on the phytoplankton reveal that these factors alone do not explain the phytoplankton dynamics in Lake Victoria [40], [41]. Therefore, the lake hydrodynamics should play the largest role in the OC4v4-*chl* decline during the stratified period. The convective circulation lasts until surface warming spreads over the whole lake. This occurs around November and is related to the onset of the OC4v4-*chl* rises in the south and the OC4v4-*chl* declines in the north (Fig. 4). The relaxation of the convective circulation and the southward tilting of the thermocline results in a northward tilting and a transitory deepening of the mixing layer in the north. The thermocline reaches an equilibrium position through repeated dampened oscillations. Support for this phenomenon is found in the measurements of thermal stratification of the water column [18]. They illustrate temporary losses of thermal discontinuity throughout the stratified period. Based on the presence of surface-type water throughout the whole water column and the rapid reappearance of thermal discontinuity, these events have been related to a tilting of the thermocline across the lake [18], [42]. Theoretical studies have reported an oscillation period of 46 days for the internal seiche motions in Lake Victoria [31]. On the other hand, field studies illustrate a regular weakening of the stratification around January [18], [33]. This weakening is also evident in the annual cycles of *NHF* and *LST* as secondary declines (Fig. 4). This feature of the annual cycle is related to the southward migration of ITCZ and the northeasterly windy season, which is characterized by weaker winds than the southeasterly windy season (Fig. S3). The secondary declines in *NHF* were particularly evident in the northern region, from where winds enter the lake. Thus, the *LST* around January in the north becomes similar or colder than that in the south (Fig. S1). Notwithstanding the lake remains stratified [18], the occurrence of short-term events of complete mixing (from the thermocline oscillation) is also more probable in the region with weaker stratification. Once the convective circulation ceases and the seasonal maxima of OC4v4-*chl* are reached, the second minor peaks of OC4v4-*chl* during the stratified period are affected by the weakening and short-term breakdown of the stratification. Therefore, the lake hydrodynamics could be related to the second peak of OC4v4-*chl*, which was greater in the southern region with respect to the northern region (Fig. 2 and 4).

Our observational record captures a year (1997–98) with a strong ENSO coupled with a positive IOD event. During this year, the alteration of the atmospheric circulation led to particularly high surface temperatures in the western Indian Ocean and shifts in the ITCZ, which brought higher precipitation to East Africa and weakening of the monsoon wind flow [43], [44]. This resulted in a notable rise in lake water level [45] and a die back of water hyacinth along the shoreline [46]. Our data show a more intense lake warming and remarkably higher annual OC4v4-*chl* (41% higher than during the other years). This appears to be related to the higher water column stability which results in an enhanced light availability for phytoplankton in the surface layer. The weakening of the surface winds was particularly evident during the northeast trade wind season (November to January; Fig. S3), which corresponded to simultaneous peaks of temperature and OC4v4-*chl* throughout the lake.

The modified dynamics observed in the El-Niño year provide a valuable opportunity to explore possible consequences of climate change. Most scenarios indicate a general weakening in wind velocity and an increase in air temperature for Eastern Africa over the coming century [47]–[48]. Furthermore, interannual variability of meteorological conditions (e.g. El-Niño events) is expected to intensify [44]. Interestingly, in nutrient-limited aquatic ecosystems such as Lake Tanganyika [49] or the ocean [6], weaker winds and/or warming conditions should reduce the vertical mixing, decrease the nutrient upwelling and diminish the phytoplankton productivity. Our analysis predicts the opposite for light limited Lake Victoria compared to nutrient limited ecosystems.

### Conclusions

The present work identifies, for the first time, key features of the basin-scale dynamics of Lake Victoria phytoplankton, demonstrating the potential uses of the remote-sensed bio-optical information to improve our understanding of the world's great lakes. Although standardised calibration algorithms are still problematic for turbid waters, the use of statistical methods based on the relative optical variability within the lake provides valuable information on central processes operating in the lakes. The robust patterns documented for Lake Victoria show that basin-scale inaccuracies in the atmospheric correction of the water surface reflected radiance may be offset by the optical variability of an extensive data series with high spatio-temporal resolution.

The correspondence between the spatio-temporal patterns and climate-related variables allowed for the development of a coherent mechanism of the observed lake-wide bio-optical variability. The linkages proposed between climate, hydrodynamics and phytoplankton agree with the findings of the previous studies [13], [18], [31]. However, new field studies are necessary to further validate and extend the basin-scale mechanism described in this work. The relationship between climate and the spatio-temporal variability of the phytoplankton biomass involve multiple ecosystem functions and services. Studies in Lake Victoria have demonstrated the relevance of the phytoplankton dynamics on organic carbon burial rates [8], physical-chemical environment [50], fish biodiversity [51] and fish yields [52]. In this regard, this study provides a novel basin-scale framework for the development of research and management strategies in Lake Victoria.

Our results show how the year to year variability in physical conditions may play a major role on phytoplankton dynamics, independent of the trend in nutrient loading. Lake managers tend to focus on phytoplankton biomass (or related variables) as an indicator of eutrophic conditions. In Lake Victoria, phytoplankton biomass was strongly controlled by annual meteorological conditions. On the other hand, the results indicate how cultural eutrophication and resulting light control on phytoplankton may change the response of aquatic ecosystems to climate warming. In recent decades, population dynamics has led to the eutrophication of many continental and coastal waters [53]. Many large lakes have experienced extensive eutrophication (e.g., Erie, Ontario, Managua, Tahoe or Mälare) or show signs of eutrophication on sub-basin scales (e.g., Biwa, Michigan) [8]. This implies that the expected ecological consequences of the trend of global warming on aquatic ecosystems should be assessed in relation to the trend of cultural eutrophication.

## Supporting Information

Figure S1 **Comparison of monthly surface temperatures (** ***LST*** **) in North and South Region.** Temporal variability of *LST* in the two regions and difference of *LST* between regions. Positive values of the difference indicate that surface layer in the north is warmer than in the south. *LST* were obtained using AVHRR data (http://podaac-www.jpl.nasa.gov/sst).
(PDF)

Figure S2 **Relationship between OC4v4-** ***chl*** **and** ***chl*** **field measurements in Lake Victoria.** Linear least-square fitting of OC4v4-*chl* against *chl* is also shown (R = 0.888, p<0.001, n = 44).
(PDF)

Figure S3 **Seasonal variability of monthly wind speed and direction in northern (A, B) and southern part (C, D) of Lake Victoria.** Vectors indicate wind direction with wind speed proportional to the length of the vector (A, C). Black vectors correspond to 1998–2004 averaged seasonal cycle and grey vectors correspond to 1997–98 El-Niño year. Wind speed is also shown as line/scatter plots (B, C). Fill circles correspond to 1998–2004 averaged seasonal cycle and open circles correspond to 1997–98 El-Niño year. Standard deviations are shown for 1998–2004 averaged seasonal cycle. Data were obtained from National Center for Environmental Prediciton (NCEP) reanalysis [30].
(PDF)

Figure S4 **Seasonal variability of** ***B*** **parameter in Lake Victoria for 1997–2004 period.** There are two offscale values, 14.4 in February-2000 and 21.9 in January-2004. The black line corresponds to the average seasonal variability for the study period. The horizontal grey line indicates *B* = 1.
(PDF)
