# Supplementary material for: Basin-Scale Control on the Phytoplankton Biomass in Lake Victoria, Africa
Source: PLoS One. 2012 Jan 9;7(1):e29962. doi: 10.1371/journal.pone.0029962 (PMC3253787; doi:10.1371/journal.pone.0029962)
Supplement: Figure S1 — Comparison of monthly surface temperatures ( LST ) in North and South Region. Temporal variability of LST in the two regions and difference of LST between regions. Positive values of the difference indicate that surface layer in the north is warmer than in the south. LST were obtained using AVHRR data (http://podaac-www.jpl.nasa.gov/sst). (PDF) [file pone.0029962.s001.pdf]

Supporting Information S1 for

Basin-scale Control on the Phytoplankton Dynamics in Lake Victoria, Africa

A. Cózar, M. Bruno, N. Bergamino, B. Úbeda, L. Bracchini, A. M. Dattilo and S. A. Loiselle

S1. Comparison of monthly surface temperatures (*LST*) in North and South Region.

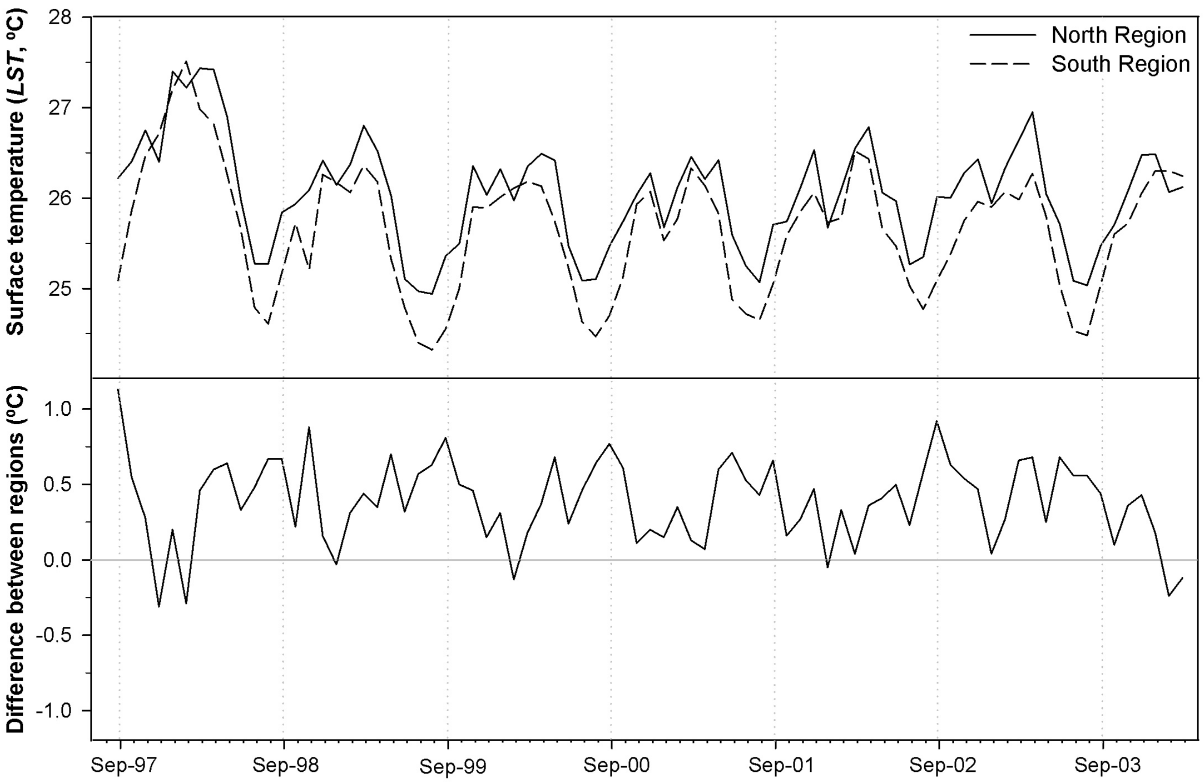

Figure S1. Comparison of monthly surface temperatures (*LST*) in North and South Region. Temporal variability of *LST* in the two regions and difference of *LST* between regions. Positive values of the difference indicate that surface layer in the north is warmer than in the south. *LST* were obtained using AVHRR data (http://podaac-www.jpl.nasa.gov/sst).
