# Supplementary material for: Basin-Scale Control on the Phytoplankton Biomass in Lake Victoria, Africa
Source: PLoS One. 2012 Jan 9;7(1):e29962. doi: 10.1371/journal.pone.0029962 (PMC3253787; doi:10.1371/journal.pone.0029962)
Supplement: Figure S2 — Relationship between OC4v4- chl and chl field measurements in Lake Victoria. Linear least-square fitting of OC4v4-chl against chl is also shown (R = 0.888, p<0.001, n = 44). (PDF) [file pone.0029962.s002.pdf]

Supporting Information S2 for

Basin-scale Control on the Phytoplankton Dynamics in Lake Victoria, Africa

A. Cózar, M. Bruno, N. Bergamino, B. Úbeda, L. Bracchini, A. M. Dattilo and S. A. Loiselle

S2. Phytoplankton control on optical environment of the Lake Victoria offshore waters.

Our analysis of the basin-scale variability of Lake Victoria assumes the control of the phytoplankton dynamics on the relative variability of the remote-sensed bio-optical environment of the offshore waters.

Previous studies have shown how the contribution of the terrigenous sources of CDOM and sediments decrease rapidly within the first 10 km from the coast [S2-1]. Spatially-extensive datasets of Lake Victoria have shown a covariance between *chl* and integrative optical properties (vertical light attenuation coefficient, Secchi depth) despite the relevant presence of additional optically-active components such as detrital particles or CDOM [S2-2].

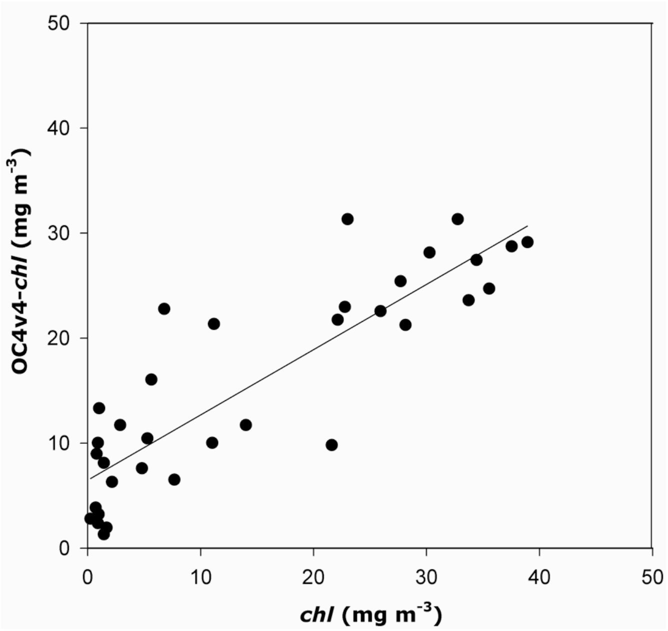

Figure S2. Relationship between OC4v4-*chl* and *chl* field measurements in Lake Victoria.

Linear least-square fitting of OC4v4-*chl* against *chl* is also shown ($R = 0.888$, $p < 0.001$, $n = 44$).

We also explored the relationship between OC4v4-*chl* and *chl* from extensive offshore data collected in Lake Victoria [S2-1, S2-3]. As it was difficult to find concurrent field data and cloud free satellite data, we compared *chl* data to weekly-average estimates of OC4v4-*chl*, introducing a small inaccuracy due to different temporal scales. The resulting correlation showed a statistically significant relationship between the variables ($R = 0.867$, $p < 0.001$, $n = 34$; Figure S2). The intercept of the ordinate shows an overestimate of the chlorophyll concentration by the OC4v4 algorithm, probably due to the relevant presence of phytoplankton-derived CDOM and detrital particles. Nevertheless, this comparison further supports the control of the phytoplankton on the relative variability of the bio-optical environment. *Chl* measurements explained 75% of the variation of OC4v4-*chl*.

References for the Supporting Information S2

S2-1. Loiselle SA, Azza N, Cózar A, Bracchini L, Tognazzi A et al. (2008) Variability in factors causing light attenuation in Lake Victoria. Fresh Biol 53: 535-545.

S2-2. Silsbe GM, Hecky RE, Guildford SJ, Mugidde R (2006) Variability of chlorophyll a and photosynthetic parameters in a nutrient saturated tropical great lake. Limnol Oceanogr 51: 2052-2063.

S2-3. LVEMP, Lake Victoria Environmental Management Programme (2002) Integrated Water Quality/ Limnology Study for Lake Victoria. Final Report. Part II: Technical Report.
