# Supplementary material for: Basin-Scale Control on the Phytoplankton Biomass in Lake Victoria, Africa
Source: PLoS One. 2012 Jan 9;7(1):e29962. doi: 10.1371/journal.pone.0029962 (PMC3253787; doi:10.1371/journal.pone.0029962)
Supplement: Figure S3 — Seasonal variability of monthly wind speed and direction in northern (A, B) and southern part (C, D) of Lake Victoria. Vectors indicate wind direction with wind speed proportional to the length of the vector (A, C). Black vectors correspond to 1998–2004 averaged seasonal cycle and grey vectors correspond to 1997–98 El-Niño year. Wind speed is also shown as line/scatter plots (B, C). Fill circles correspond to 1998–2004 averaged seasonal cycle and open circles correspond to 1997–98 El-Niño year. Standard deviations are shown for 1998–2004 averaged seasonal cycle. Data were obtained from National Center for Environmental Prediciton (NCEP) reanalysis [30]. (PDF) [file pone.0029962.s003.pdf]

Supporting Information S3 for

Basin-scale Control on the Phytoplankton Dynamics in Lake Victoria, Africa

A. Cózar, M. Bruno, N. Bergamino, B. Úbeda, L. Bracchini, A. M. Dattilo and S. A. Loiselle

S3. Monthly wind speed and direction in Lake Victoria.

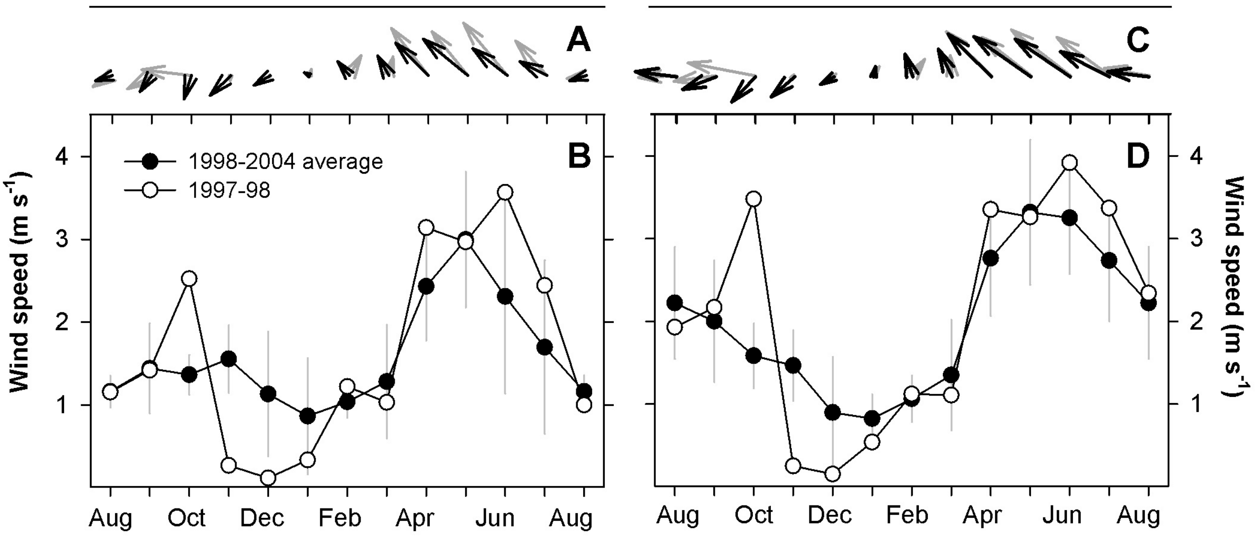

Figure S3. Seasonal variability of monthly wind speed and direction in northern (A, B) and southern part (C, D) of Lake Victoria. Vectors indicate wind direction with wind speed proportional to the length of the vector (A, C). Black vectors correspond to 1998-2004 averaged seasonal cycle and grey vectors correspond to 1997-98 El-Niño year. Wind speed is also shown as line/scatter plots (B, C). Fill circles correspond to 1998-2004 averaged seasonal cycle and open circles correspond to 1997-98 El-Niño year. Standard deviations are shown for 1998-2004 averaged seasonal cycle. Data were obtained from National Center for Environmental Prediciton (NCEP) reanalysis [30].
