# Supplementary material for: Basin-Scale Control on the Phytoplankton Biomass in Lake Victoria, Africa
Source: PLoS One. 2012 Jan 9;7(1):e29962. doi: 10.1371/journal.pone.0029962 (PMC3253787; doi:10.1371/journal.pone.0029962)
Supplement: Figure S4 — Seasonal variability of B parameter in Lake Victoria for 1997–2004 period. There are two offscale values, 14.4 in February-2000 and 21.9 in January-2004. The black line corresponds to the average seasonal variability for the study period. The horizontal grey line indicates B = 1. (PDF) [file pone.0029962.s004.pdf]

Supporting Information S4 for

Basin-scale Control on the Phytoplankton Dynamics in Lake Victoria, Africa

A. Cózar, M. Bruno, N. Bergamino, B. Úbeda, L. Bracchini, A. M. Dattilo and S. A. Loiselle

S4. Relative influence of convective motion and wind-induced currents on the lake-wide circulation pattern.

The relative strength of convection with respect to wind-induced currents was evaluated by means of the dimensionless parameter B [S4-1]:

$$B = \left| \frac{u_*^2 L}{\beta \Delta T_w g h^2} \right|$$

where $u_* = (\tau / \rho_w)^{1/2}$ is the surface shear velocity; $\tau = \rho_a C_d W^2$ is the wind stress at the water surface; ρ_w and ρ_a are the densities of water and air respectively; C_d is the drag coefficient at the water surface; W is the wind speed at 10 m height; L is the length of the lake; h is the thickness of the convective cell; ΔT_w is the difference between the surface water temperature at either end of the lake; β is the coefficient of thermal expansion of water at 25ºC ($2.5 \cdot 10^{-4}$ ºK^{-1}); and g is the gravitational acceleration. Lake-wide circulation is dominated by convection for $B < 1$, while wind-induced currents dominate for $B > 1$.

We use monthly water temperature estimated from AVHRR data (*LST*, Supporting Information S1), monthly wind speed obtained from the NCEP reanalysis data (Supporting Information S3), a lake length of 200 km, and a convective cell depth of 20 m. Note that a greater thickness of the convective cell would result in a lower B estimates.

The results show that B was generally less than 1 between August and November (Figure S4). Particularly low values were reached in August and September. For $B < 1$, the strength of the wind is insufficient to overcome the lake-wide convective circulation induced by the horizontal temperature gradient.

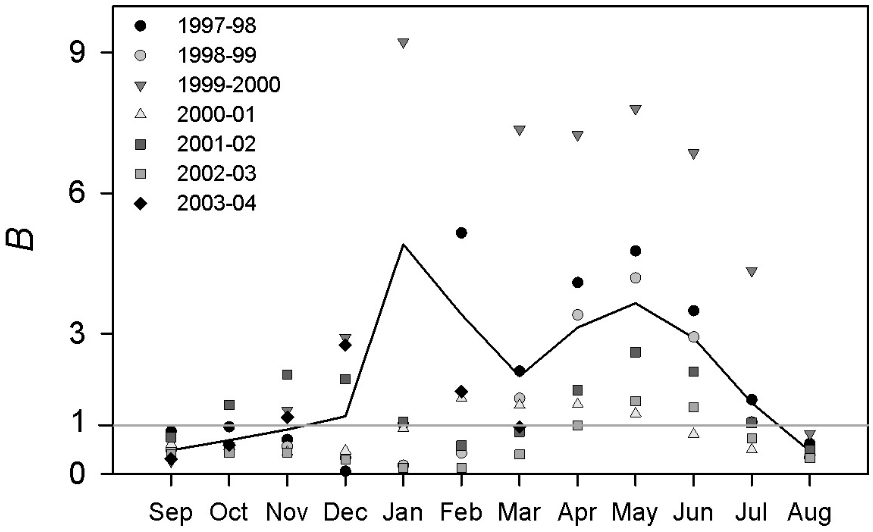

Figure S4. Seasonal variability of *B* parameter in Lake Victoria for 1997-2004 period. There are two offscale values, 14.4 in February-2000 and 21.9 in January-2004. The black line corresponds to the average seasonal variability for the study period. The horizontal grey line indicates *B* = 1.

Reference for the Supporting Information S4

S4-1. Cormack DE, Stone GP, Leal LG (1975) The effect of upper surface conditions on convection in a shallow cavity with differentially heated end-walls. Int. J. Heat Mass Transf. 18: 635–648.
